# Evidences of grain boundary capacitance effect on the colossal dielectric permittivity in (Nb + In) co-doped TiO_2_ ceramics

**DOI:** 10.1038/srep08295

**Published:** 2015-02-06

**Authors:** Jinglei Li, Fei Li, Chao Li, Guang Yang, Zhuo Xu, Shujun Zhang

**Affiliations:** 1 Electronic Materials Research Laboratory, Key Laboratory of the Ministry of Education and International Center for Dielectric Research, Xi'an Jiaotong University, Xi'an 710049, China; 2 Materials Research Institute, Pennsylvania State University, University Park, Pennsylvania 16802, USA

## Abstract

The (Nb + In) co-doped TiO_2_ ceramics were synthesized by conventional solid-state sintering (CSSS) and spark plasma sintering (SPS) methods. The phases and microstructures were studied by X-ray diffraction, Raman spectra, field-emission scanning electron microscopy and transmission electron microscopy, indicating that both samples were in pure rutile phase while showing significant difference in grain size. The dielectric and I–V behaviors of SPS and CSSS samples were investigated. Though both possess colossal permittivity (CP), the SPS samples exhibited much higher dielectric permittivity/loss factor and lower breakdown electric field when compared to their CSSS counterparts. To further explore the origin of CP in co-doped TiO_2_ ceramics, the I–V behavior was studied on single grain and grain boundary in CSSS sample. The nearly ohmic I–V behavior was observed in single grain, while GBs showed nonlinear behavior and much higher resistance. The higher dielectric permittivity and lower breakdown electric field in SPS samples, thus, were thought to be associated with the feature of SPS, by which reduced space charges and/or impurity segregation can be achieved at grain boundaries. The present results support that the grain boundary capacitance effect plays an important role in the CP and nonlinear I–V behavior of (Nb + In) co-doped TiO_2_ ceramics.

Materials with colossal permittivity (CP, i.e., relative dielectric permittivity is larger than 1000) are in the focus of interest, which will benefit the smaller and faster electronics. Recently, remarkable dielectric behavior was reported in (Nb + In) co-doped TiO_2_^1^,^2^,^3^, which possessed colossal dielectric permittivity (>10^4^) as well as low dielectric loss (<0.05) over wide temperature range of 80 ~ 450 K, showing the possibility to replace the state-of-the-art CP materials, such as CaCu_3_Ti_4_O_12_^4^,^5^,^6^ and doped-BaTiO_3_^7^,^8^,^9^.

In order to further improve the property of (Nb + In) co-doped rutile TiO_2_ for potential capacitor applications, it is desirable to clarify the related CP mechanism. A new mechanism was proposed by Hu *et al*,^1^ who first reported CP and low loss properties in co-doped TiO_2_, i.e., the electron-pinned defect-dipoles. On the other hand, Li *et al.*^2^ showed that the grain boundary capacitance (GBC) effect may significantly contribute to the CP of (Nb + In) co-doped TiO_2_ ceramics, based on the impedance spectroscopy. However, it is known that impedance spectroscopy cannot be used as the direct and solid evidences to exclude or include the GBC effect. For example, the two semicircles in the impedance spectroscopy are generally used to analyze the GBC effect in dielectrics, while this phenomenon has multiple origins. In general, dielectrics consist of two heterogeneous regions may show two semicircles in impedance spectroscopy, i.e., GBs in ceramics, electrode depletion zones^10^, phase separation^11^, nonstoichiometric surface layers^12^,^13^ and even dirt on the surface^14^ are the possible origins. From intrinsic respect, on the other hand, semicircles in impedance spectroscopy will also show up when charge-transport is present.^15^,^16^ Therefore, in order to verify the contribution of GBC effect to the CP of (Nb + In) co-doped TiO_2_, more solid evidences are still required besides the impedance spectroscopy.

In the present paper, we provided two experimental approaches, by which the CP mechanism of (Nb + In) co-doped TiO_2_ was explored. Firstly, the (Nb + In) co-doped TiO_2_ ceramics were synthesized by two different approaches, i.e., conventional solid-state sintering (CSSS) and spark plasma sintering (SPS). The main feature of SPS is the fast heating rates (600°C/min) due to Joule heating^17^. Compared with conventional sintering, SPS method can be used to make the sintered ceramics more homogeneous and achieve cleaner grain boundaries (reduced impurity segregation and secondary phase), and thus may significantly reduce the grain boundary resistivity^17^,^18^. Synthesizing the co-doped TiO_2_ ceramics by CSSS and SPS techniques allows us to analyze the dielectric properties of co-doped TiO_2_ ceramics with different type of GBs. Secondly, the co-doped TiO_2_ ceramics sintered by CSSS technique were coated with micro-electrode by lithographic process, where the electrode is smaller than the grain, allowing us to analyze the I–V behaviors for single grain and GB. Based on these experiments, the properties of GB could be further explored and the role of GBC effect on the CP behavior of (Nb + In) co-doped TiO_2_ could be clarified.

## Results and Discussion

**Figure 1:**
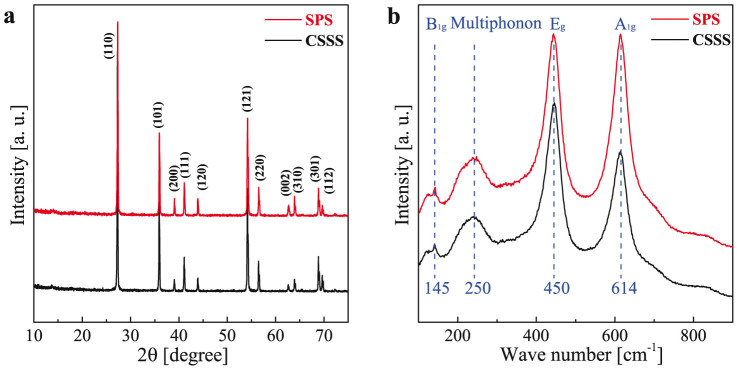
Determination of the phase structure for 1 mol% (Nb + In) co-doped TiO_2_ ceramics prepared by CSSS and SPS. (a) The XRD patterns. The lattice indexes of rutile phase are given according to the standard XRD card. The XRD patterns show that both ceramics are crystallized in a rutile phase. (b) Raman spectra of the ceramics. The dotted lines (145 cm^−1^, 250 cm^−1^, 450 cm^−1^, and 614 cm^−1^) indicate the Raman vibration mode of rutile phase. The dotted line around 250 cm^−1^ is a multi-phonon peak.

The X-ray powder diffraction results for SPS and CSSS ceramics are shown in Fig. 1 (a), revealing that both samples are in pure rutile phase. Fig. 1 (b) shows the Raman spectra of the ceramics. There are four Raman active fundamental modes in rutile TiO_2_: B_1g_ (143 cm^−1^), E_g_ (447 cm^−1^), A_1g_ (612 cm^−1^), and B_2g_ (826 cm^−1^)^19^. The 143 cm^−1^ (B_1g_) Raman peak is an O-Ti-O bond bending mode, the 612 cm^−1^(A_1g_) relates to Ti-O stretch mode while the 447 cm^−1^ (E_g_) mode is due to oxygen atom liberation along the c-axis out of phase^20^. The 614 cm^−1^, 450 cm^−1^ and 145 cm^−1^ Raman peaks in Fig. 1 (b) can be assigned to be the 612 cm^−1^, 447 cm^−1^ and 143 cm^−1^ lines of the pure TiO_2_, respectively^21^, again demonstrating that the doped TiO_2_ ceramics by CSSS and SPS are in rutile phase. It should be noted that the peak around 250 cm^−1^ of Raman spectra is not due to one of the four Raman-active zone-center (*k* = 0) phonons of the rutile structure^22^. It is generally believed that the peak around 250 cm^−1^ is multiphonon peak caused by second-order Raman scattering in rutile structure^23^.

**Figure 2:**
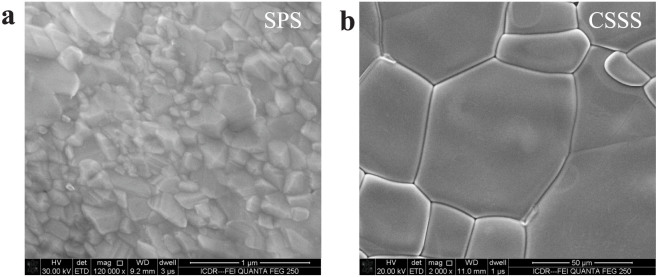
The SEM images for 1 mol% (Nb + In) co-doped TiO2 ceramics. (a) and (b) are SEM images for SPS and CSSS samples, respectively, where the grains and GBs can be observed. The voltages of SEM image observation for SPS and CSSS samples are 30 kV and 20 kV, respectively. The work distances of SEM image observation for SPS and CSSS samples are 9.2 mm and 11.0 mm, respectively.

**Figure 3:**
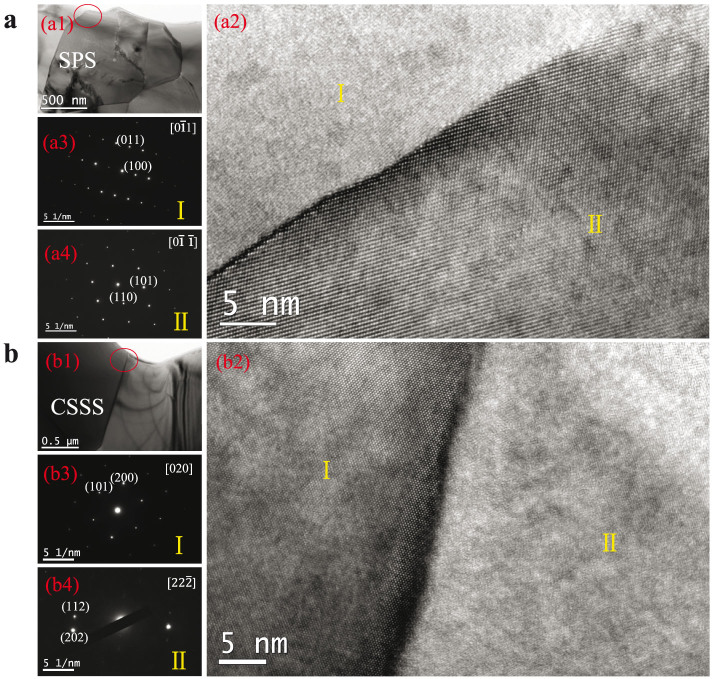
The TEM images for 1 mol% (Nb + In) co-doped TiO2 ceramics prepared by (a) SPS and (b) CSSS. (a1) and (b1) are the plane-view TEM image for SPS and CSSS samples, respectively. (a2) and (b2) are the high-resolution TEM image of red circle area in each plane-view. In the region I of (a2) and region II of (b2), alternately dark and bright stripes are vague, which is due to the fact that the incident electron beam is not strictly along the specific zone axis. (a3) and (a4) are the Selected Area Electron Diffraction (SAED) spots for the marked sides of grain boundary shown in (a2). The SAED spots of (a3) and (a4) are recorded along the 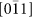 and 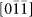 zone axis, respectively. (b3) and (b4) are the SAED spots for the marked side of grain boundary shown in (b2). The SAED spots of (b3) and (b4) are recorded along the [020] and 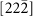 zone axis, respectively. The lattice planes in each SAED pattern were given according to the d-spacing of rutile TiO2.

Fig. 2 presents the SEM pictures for the (a) SPS and (b) CSSS samples. For the SPS sample, uniform grain size and denser microstructure can be obtained. The average grain size of the SPS ceramics is around 100–300 nm as illustrated in Fig.2 (a). Fig. 2 (b) shows SEM picture of CSSS sample, where significantly larger grain size (5–100 μm) was observed when compared to that of SPS sample. The grain size of SPS sample was found to be much smaller than that of CSSS sample, which is due to the feature of SPS technique, i.e., fast heating rate and short dwelling time, leading to short time for grain growth. For CSSS technique, on the contrary, larger grain size was obtained due to the much longer dwelling time at sintering temperature^24^,^25^. Figs. 3 (a) and (b) show the high resolution TEM images for SPS and CSSS samples, respectively. For CSSS sample, although the overlapped region^26^ existed at the boundary plane, no glassy phase was observed. In contrast to the general recognition of grain boundary thickness (a few nanometers), the grain boundary of SPS sample is very thin and it is hard to estimate the thickness. In addition, the existence of liquid phase or other secondary phases at GBs are uncertain for both samples, since the selected TEM regions are very small compared to the whole samples and the glassy materials may also be removed from the samples during ion milling process.

**Figure 4:**
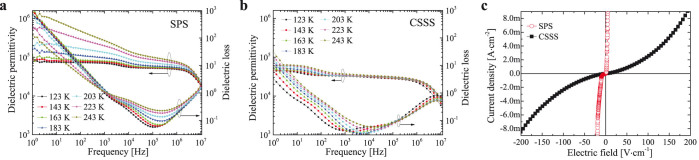
The dielectric behavior and current density-electric field (I–V) curves of 1 mol% (Nb + In) co-doped TiO_2_. Dielectric permittivity of (a) SPS and (b) CSSS samples with respect to frequency at various temperatures; (c) current density-electric field (I–V) characteristics for SPS and CSSS samples, measured at 25°C.

Fig. 4 shows dielectric permittivity and loss as a function of frequency for (a) SPS and (b) CSSS samples. At temperature of 123 K, it is clear that two dielectric plateaus exist in 1 Hz–1 kHz and 1 kHz–1 MHz frequency ranges for both samples, being followed by a large drop of the values. In general, the relaxation behavior over frequency range of 1 Hz–1 kHz can be attributed to the additional interfaces between electrode and sample^27^,^28^, while the origin of the relaxation behavior over frequency of 1 kHz-1 MHz is controversial, including mechanisms of charge-density wave formation^29^,^30^,^31^,^32^, hopping charge transport^33^,^34^,^35^,^36^, metal-insulator transition^37^,^38^,^39^,^40^,^41^, various kinds of interface effects^42^,^43^,^44^,^45^,^46^, and etc. The low frequency (1 Hz < *f* < 1 kHz) dielectric permittivity of SPS ceramics (~80000) was found to be much higher than that of CSSS samples (~42000), meanwhile possessing higher dielectric loss. For intermediate frequency (1 kHz < *f* < 1 MHz) dielectric plateau, dielectric permittivity of SPS sample is on the order of ~70000, which is also much higher than that of CSSS sample (~30000). With increasing the temperature, dielectric plateaus of both samples shift slightly to higher frequency region, revealing that the relaxation is related to the thermally activated process^47^. Fig. 4 (c) presents the I–V characteristics for CSSS and SPS ceramics, where nonlinear I–V behavior was observed for both samples. Of particular interest is that the current density of SPS sample is much higher than that of CSSS counterpart, showing a very low breakdown electric field of ~10 V/cm. According to Fig. 2 and Fig. 3, the different dielectric and I–V behaviors between CSSS and SPS samples should be attributed to their different microstructures.

**Figure 5:**
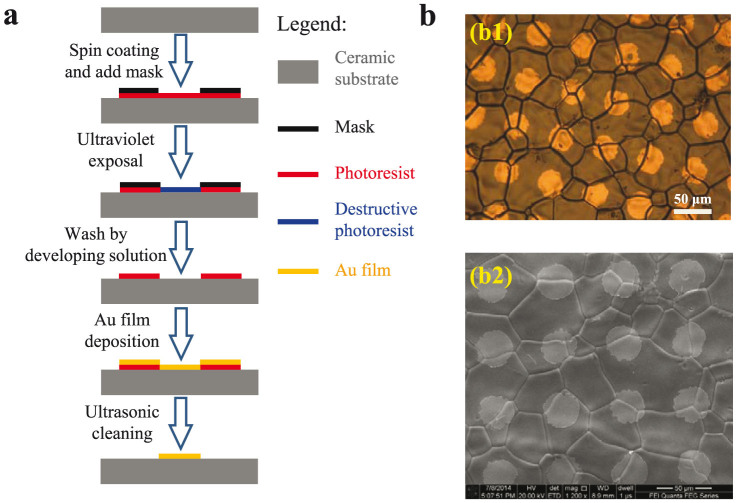
Flow-chart of preparing the sample for micro-contact measurement. (a) The lithographic procedure, where the figure legends for different materials were given in the right. The detailed description was shown in *Method* section. (b) The optical microscopic (OM) (b1) and SEM (b2) images of the surface morphology of the CSSS sample with micro-electrodes.

**Figure 6:**
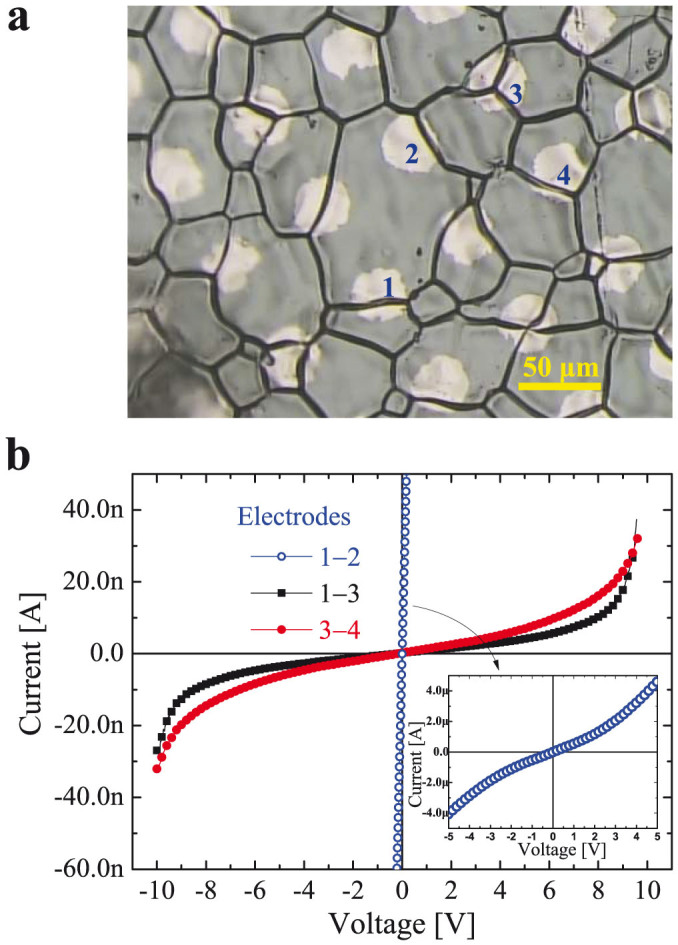
Micro-contact I–V characteristics within single grain and across individual grain boundaries for 1 mol% (Nb + In) co-doped TiO_2_ ceramics. (a) The OM image of CSSS sample with micro-electrodes, where the measured points (micro-electrodes) on the sample are marked. (b) I–V characteristics between different pairs of micro-electrodes. The inset indicates the I–V behavior within a single grain up to ~5.0 V.

To further explore the controlling factors responsible for the difference presenting in I–V and dielectric responses, the gold micro-electrodes were deposited on the surface of CSSS samples by lithographic process to perform micro-contact analysis, as given in Fig. 5. The detailed information of sample preparation was presented in *Method* section. Fig. 6(a) shows the patterned microelectrodes and measured points on the surface of the CSSS sample. Fig. 6(b) gives the measured I–V curves across the grain boundaries, and the curves measured within a grain as comparison. In contrast to the nearly ohmic behavior between electrodes 1 and 2 within a grain, the I–V curves measured across grain boundaries (1–3 or 3–4) are nonlinear with a threshold voltage of ~7.0 V, indicating that the charge transfer among grains is blocked by the GB. According to previous XPS studies^1^,^2^, high conductivity of grains was attributed to the reduction of Ti^4+^ ions to Ti^3+^ ions. Before the dielectric breakdown, GBs show much higher resistance when compared with grains, because of which the colossal dielectric permittivity in co-doped TiO_2_ ceramics can be explained by the GBC model. According to the brick network model and I–V curves in Fig. 4(c), the blocking effect of GBs in SPS sample should be much lower than that in CSSS sample. As a result, the higher dielectric permittivity and loss factor in SPS ceramics can be attributed to the weaker blocking effect of GBs. It is generally accepted that the blocking effect of GBs is due to the different chemical potential between the grain and GB, which can be changed by dopants segregation, GB thickness, secondary phases, space charge density, etc. in GBs^48^. Based on the feature of SPS, the reduced space charge and/or impurity segregation at GBs was thought to be an possible reason for the weak blocking effect in SPS sample^17^,^18^,^49^. It was reported that SPS method could be used to effectively reduce the GB resistivity, owing to the effect of electrical discharges among the grains when the high current passed through the graphite die during SPS^18^. On the other hand, it should be noted here that the exact reason for the weak blocking effect in SPS cannot be explored according to the present experiments. Some other factors may also be responsible for the low level of blocking effect of GB in SPS samples. For example, the small grain size of SPS samples may be an important factor for the low blocking effect of GB. The conductivity of GB was found to rapidly increase with decreasing the grain size in doped zirconia ceramics, which was attributed to the reduced GB space charge potential in small-grain-size-ceramics^50^,^51^.

In summary, 1 mol% (Nb + In) co-doped TiO_2_ ceramics were fabricated by CSSS and SPS for analyzing the CP mechanism. The I–V behavior across individual GB as well as within single crystalline grains was directly measured in CSSS samples by micro-contact analysis, showing that the GBs serve as obstacles to the current flow through the conductive bulk grains. According to the I–V behaviors of SPS and CSSS samples, the blocking effect of GB was much lower in SPS samples than that in the CSSS samples, which was thought to be associated with the feature of SPS method (reduced impurity segregation and/or space charges can be achieved at grain boundaries by SPS technique). As a result, higher dielectric permittivity and loss factor were observed in SPS ceramics. The results in this research support that the GBC effect plays an important role in nonlinear I–V and CP phenomena in (Nb + In) co-doped TiO_2_ ceramics.

## Methods

### Sample preparation

The 1 mol % (Nb + In) co-doped TiO_2_ ceramics were synthesized using both CSSS and SPS methods, where the rutile TiO_2_ (99.99%), Nb_2_O_5_ (99.99%) and In_2_O_3_ (99.99%) powders were used as raw materials. The weighed powder were added into a ball-milling jar, and milled for 24 h in distilled water with Y-stabilized ZrO_2_ media. After drying, the powders were then divided into two sets. One set of powders with addition of 5 wt% polyvinyl alcohol (PVA) was uniaxial pressed into disks at a pressure of 10 MPa in a 10 mm diameter stainless steel cylindrical die, then binder burn-out at 600°C for half hour and sintered in alumina crucible with 3°C/min heating rate by the CSSS method at 1450°C for 20 h. Another set of powders was sintered by SPS technique using a Dr. Sinter 2050 SPS apparatus (Syntex Inc., Kanagawa, Japan) at 600°C for 5 min with a pressure of 300 MPa and 1150°C for 3 min with a pressure of 60 MPa. For SPS, the powders were loaded into a graphite die, with inner diameter of 12 mm. Graphite foil was placed between the powders and die to prevent the possible adhesion. After cold-pressing, the die was placed in the SPS apparatus. The temperature, measured using a thermocouple inserted in the graphite die, was raised to 600°C in 2 min and then to the sintering temperature at ~300°C/min. The temperature inside the die was generally higher than that measured by the thermocouple. Calculations and previous experimental observations suggest that the temperature difference was less than 150°C in the temperature range of interest^52^. After holding at sintering temperature for a fixed period, samples were cooled naturally after removing the current and releasing the pressure. Any graphite foil that adhered to pellet surfaces was removed. Pellets were subsequently heated at 800°C for 6 h in a muffle furnace to oxidize residual graphite prior to further characterization. The CSSS and SPS samples with conducting silver paste were fired at 600°C for 0.5 h for a good contact to test the dielectric permittivity and loss as a function of frequency. For SEM investigation, CSSS samples were polished and thermally etched at 1400°C for 10 min with a heating/cooling rate of 5°C/min, then was deposited with Au film by rate of 6 Å/s for 10 s. The cross-section of SPS sample was directly used for SEM investigation. The TEM specimens of both CSSS and SPS were prepared by mechanical grinding, polishing and dimpling followed by argon ion milling.

### Lithographic process

The flow-chart for lithographic process is shown in Fig. 5. The detailed process is described as follows. The sample used as the ceramic substrate was first put into an ultrasonic cleaning bath to make the surface clean in acetone, alcohol and de-ionized water. And then the ceramic substrate with clean surface was deposited with the positive photo-resist by spin coating, which was intimately patterned with a molybdenum (Mo) mask. The mask used in this work was fabricated by photochemistry etching technique with thickness of 0.2 μm, consisting of a 30-μm-diameter cavity array with a 30-μm period distance. The ceramic substrate with photo-resist and mask was then exposed to ultraviolet light to destruct the molecular chain of photo-resist in the latent image region, where it would be washed by the developing solution. Next, we evaporated a uniform layer of Au ~7.2 nm (6 Å/s) on the surface of ceramic substrate with cavity photo-resist. Finally, the prepared sample was again ultrasonic cleaned in acetone solution for 20 minutes to wash away the leftover photo-resist. The above whole lithographic procedure is shown in Fig. 5 (a). Fig. 5 (b) presents the surface morphology of the obtained sample with micro-electrodes.

### Characterization

The phase of samples was characterized by X-ray diffraction (XRD, D/MAX 2400, Japan) and Raman spectra (Raman, HR800, France). The Raman spectrum was obtained with 541.32 nm Ar^+^ laser as an excitation source. The microstructure was investigated by a field-emission scanning electron microscopy (FE-SEM/EDX, Quanta F250, USA) and a transmission electron microscopy (TEM, JEM 2100, Japan). The dielectric properties of the ceramics were measured by a Novo-control broadband dielectric spectrometer with an alpha-A high performance frequency analyzer over frequency range of 10^−1^–10^7^ Hz and temperature range of 125–475 K. For the micro-contact I–V measurements, low contact resistance tungsten microprobes (10 μm in diameter) were used in a manual probe station (SUMMIT 11862B, Cascade, USA). The currents flow between individual grains and within grains were measured using a semiconductor characterization system (4200-SCS/F, Keithley, USA) connected to the probe station, which was also used to measure I–V characteristics for CSSS and SPS ceramics.

## Author Contributions

The project was conceived and designed by F.L., Z.X. and S.J.Z., J.L.L. and F.L. performed the experiments; Z.X. assisted in the measurement of dielectric property; C.L. and G.Y. assisted in the measurement of TEM; J.L.L., F.L. and S.J.Z. prepared the manuscript. All authors discussed the results and contributed to the refinement of the paper.
